# P-275. Risk Factors for Community Colonization with Extended-Spectrum Cephalosporin-Resistant *Enterobacterales* (ESCrE) among People with HIV in Botswana

**DOI:** 10.1093/ofid/ofae631.479

**Published:** 2025-01-29

**Authors:** Bogadi Loabile, Ebbing Lautenbach, Naledi B Mannathoko, Mosepele Mosepele, Mosepele Mosepele, Ashley R Styczynski, Rachel Mann Smith, Leigh Cressman, Anne Jaskowiak, Warren B Bilker, Kevin Alby, Laurel Glaser, Melissa Richard-Greenblatt, Laura Cowden, Kgotlaetsile Sewawa, Dimpho Otukile, Giacomo Paganotti, Margaret Mokomane, Robert Gross

**Affiliations:** University of Pennsylvania, Philadelphia, Pennsylvania; University of Pennsylvania, Philadelphia, Pennsylvania; University of Botswana, Gaborone, South-East, Botswana; University of Botswana, Gaborone, South-East, Botswana; University of Botswana, Gaborone, South-East, Botswana; Centers for Disease Control and Prevention, Atlanta, GA; CDC, Atlanta, Georgia; University of Pennsylvania Perelman School of Medicine, Philadelphia, Pennsylvania; University of Pennsylvania, Philadelphia, Pennsylvania; University of Pennsylvania Perelman School of Medicine, Philadelphia, Pennsylvania; University of North Carolina, Chapel Hill, North Carolina; University of Pennsylvania, Philadelphia, Pennsylvania; Hospital for Sick Children, toronto, Ontario, Canada; University of Pennsylvania, Philadelphia, Pennsylvania; Botswana-University of Pennsylvania Partnership, Gaborone, South-East, Botswana; University of Botswana, Gaborone, South-East, Botswana; Botswana- University of Pennsylvania Partnership, Gaborone, South-East, Botswana; University of Botswana, Gaborone, South-East, Botswana; University of Pennsylvania, Philadelphia, Pennsylvania

## Abstract

**Background:**

Extended-spectrum cephalosporin-resistant Enterobacterales (ESCrE) are a major global threat, and there is a significant gap in research on the burden and associated risk factors for ESCrE in low-and middle income countries(LMICs). This is particularly true for people with HIV (PWH), who make up a significant proportion of the population in sub-Saharan Africa. In Botswana, 20% of individuals aged 15-49 are PWH. The risk factors associated with ESCrE colonization, typically a precursor to infection, are important to understand as such infections can result in increased healthcare costs as well as high morbidity and mortality.

**Figure:**
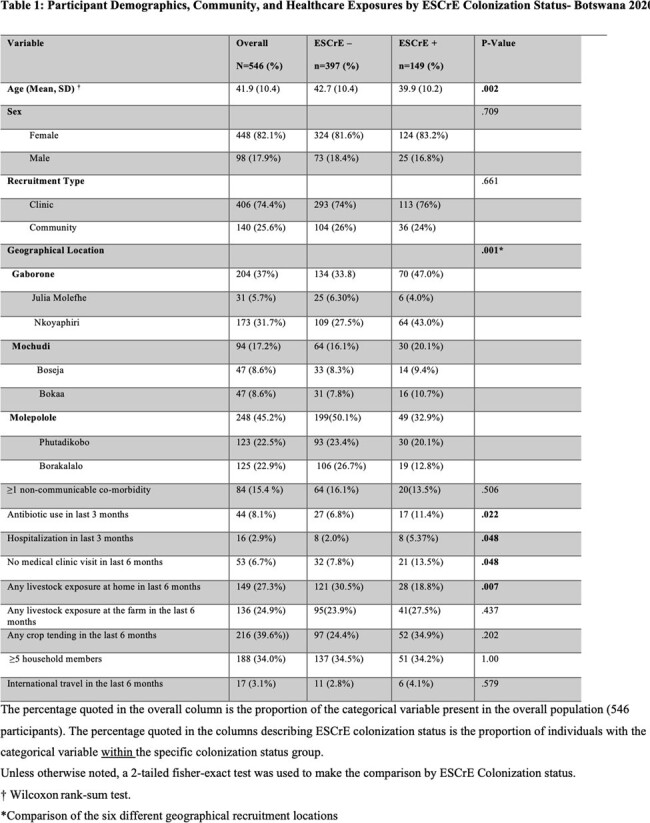

**Methods:**

Within a larger regional surveillance study, 546 adults with HIV were recruited from clinics and communities in 3 districts and underwent interviews and rectal sampling. ESCrE was defined as Enterobacterales demonstrating non-susceptibility to ceftriaxone or ceftazidime.

**Figure:**
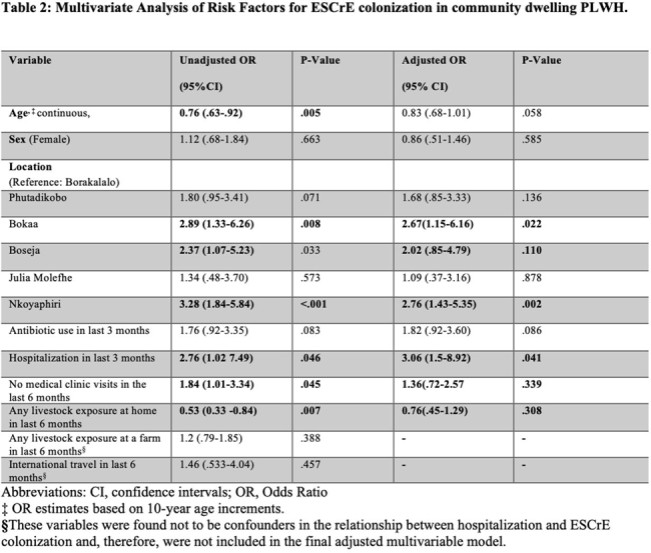

**Results:**

27% of participants screened positive for ESCrE colonization. The mean CD4 count was 635 cells/mm3 (SD± 267). Table 1 describes the demographics of the participants with and without ESCrE colonization. *Escherichia coli* was the most commonly isolated ESCrE (146/174; 84%). Bivariate and multivariate analysis was used to determine risk factors associated with ESCrE colonization (Table 2). Recent hospitalization and certain geographic locations were independent risk factors for ESCrE colonization. Recent antibiotic use had an elevated OR for ESCrE colonization that did not achieve statistical significance in adjusted analysis.

**Conclusion:**

These results add to the limited data on risk factors associated with ESCrE colonization in PWH. Hospitalization is an independent risk factor for colonization despite controlling for antibiotic use, which suggests the need for further investigation into hospital-specific factors that contribute to ESCrE colonization. Further research is needed to understand the geographic differences in ESCrE colonization in this setting. As LMICs with high HIV burdens build capacity for antimicrobial stewardship and infection prevention infrastructure in healthcare, research on unique potential mechanisms that result in multi-drug resistant colonization in PWH may impact strategies and priorities to combat antimicrobial resistance.

**Disclosures:**

**Robert Gross, MD, MSCE**, Pfizer Inc: DSMB member for drug unrelated to study

